# Systemic lupus erythematosus complicated with Fanconi syndrome: a case report and literature review

**DOI:** 10.3389/fped.2023.1230366

**Published:** 2024-01-05

**Authors:** Lili Lou, Hui Guo, Meiying Shao

**Affiliations:** ^1^ Department of Pediatric Pulmonology and Immunology, West China Second University Hospital, Sichuan University, Chengdu, China; ^2^ Key Laboratory of Birth Defects and Related Diseases of Women and Children (Sichuan University), Ministry of Education, Chengdu, China; ^3^ Department of Pediatrics, West China Second University Hospital, Sichuan University, Chengdu, Sichuan, China; ^4^ West China School of Public Health and West China Fourth Hospital, Sichuan University, Chengdu, Sichuan, China

**Keywords:** atypical, childhood systemic lupus erythematosus, lupus nephritis, renal tubular acidosis, Fanconi syndrome

## Abstract

**Background:**

Systemic lupus erythematosus is an autoimmune disease with diverse clinical manifestations. The symptoms of SLE in children are more atypical than adults. Childhood SLE complicated with Fanconi syndrome is extremely rare and even more difficult to diagnose.

**Case presentation:**

This article reports a preschool boy with SLE who presented with renal tubular acidosis, accompanied by weakness in both lower limbs, delayed growth, and malnutrition. It was later found that the patient had the complication of Fanconi syndrome with renal tubular acidosis. Ultimately, renal biopsy confirmed lupus nephritis. The patient was treated with corticosteroid combined with mycophenolate mofetil, hydroxychloroquine, and belimumab. The symptoms of the child were relieved.

**Conclusion:**

Here we report an extremely rare case of childhood SLE complicated with Fanconi syndrome. There has been no similar clinical report. It is necessary to be alert to the possibility of atypical SLE in children to avoid missed diagnosis and misdiagnosis.

## Introduction

Systemic lupus erythematosus (SLE) is an autoimmune disease that involves multiple organs and systems throughout the body. Multiple auto-antibodies, represented by antinuclear antibodies (ANA), exist in SLE patients, which can cause irreversible damage to affected organs. Lupus nephritis (LN) is the most common and important complication of SLE, and also one of the main causes of death in patients with SLE. In recent years, a large number of studies have shown that the pathogenesis of childhood-onset SLE (cSLE) is the result of the interaction among genetic, immunological, racial, and environmental factors. The prevalence rate of cSLE is approximately 3.3–24 per 100,000, while only 10%–20% of the patients are diagnosed during childhood (1, 2). The clinical manifestations of cSLE are diverse, with 60%–85% of children having atypical onset symptoms, and some children only showing slight abnormalities in urine routine tests in the early stage, which poses a difficulty and interference to clinical diagnosis (3, 4). Childhood lupus nephritis often involves the glomerulus, renal tubules, and interstitium, while the involvement of the latter two is rare (5). Compared with adult SLE, cSLE progresses more rapidly, has a wider range of involvements, is more severe, and has a poorer prognosis and a higher incidence of renal damage (1). About 5%–22% of children with LN will develop end-stage renal disease (ESRD) within 5–10 years (6–9). Therefore, early diagnosis and treatment are crucial for reducing organ damage, improving prognosis, and reducing mortality. This article reports a boy with preschool onset of SLE, with presenting symptoms of renal tubular acidosis such as fatigue, polyuria, polydipsia, and delayed growth and development. During the course of the disease, the complication of Fanconi syndrome was found. Except for positive auto-antibodies, there were no typical lupus symptoms such as fever, rash, alopecia, arthritis, and oral ulcers. Finally, renal biopsy confirmed lupus nephritis. Therefore, this case of cSLE had extremely atypical clinical manifestations.

## Case presentation

**Figure 1:**
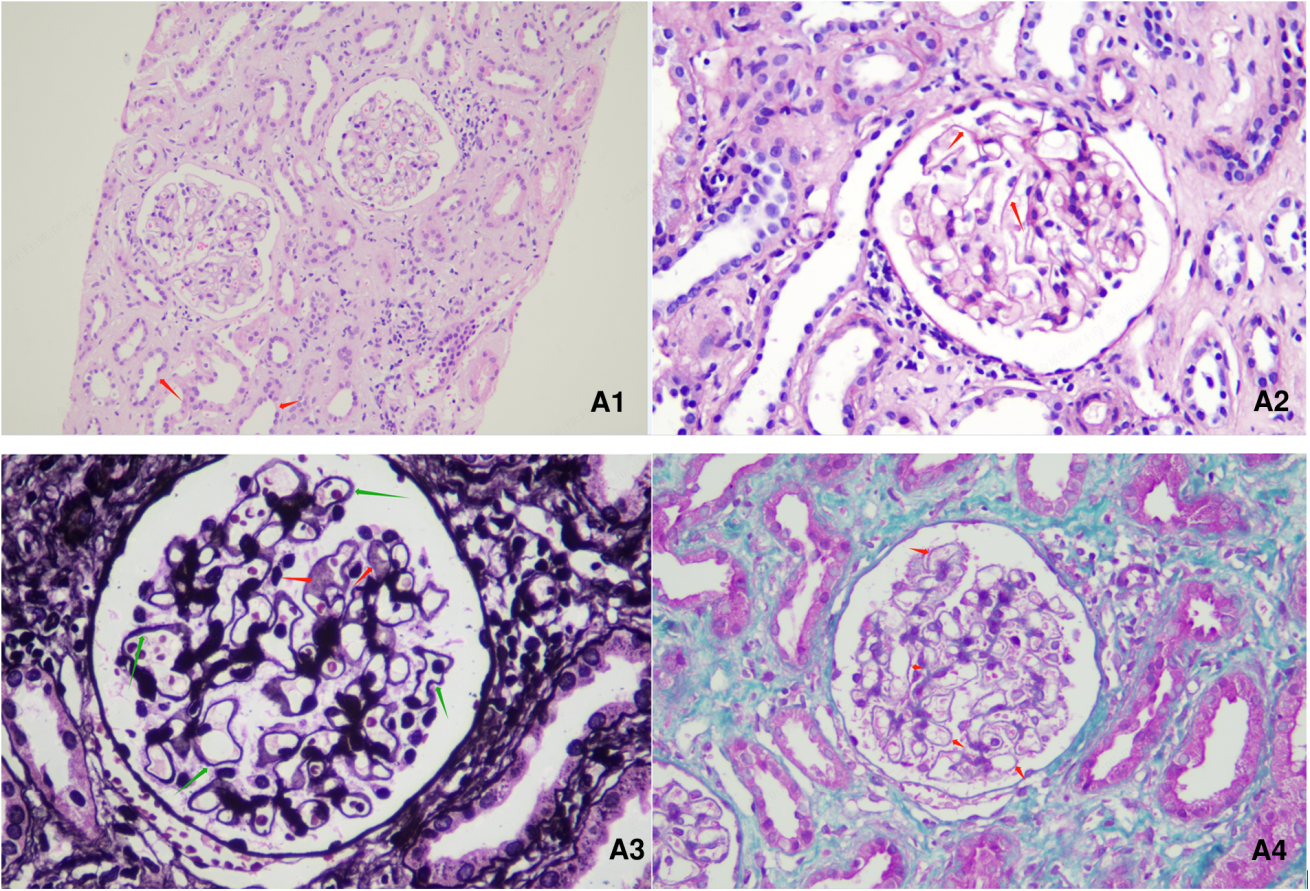
Renal pathological presentation of the patient. (A1): HE (hematoxylin-eosin) staining, ×200 magniﬁcation. (A2): PAS (periodic Acid Schiff), staining, ×400 magniﬁcation. (A3): PASM (periodic acid-silver methenamine) staining, ×600 magniﬁcation. (A4): Masson staining, ×400 magniﬁcation. Among the 4 glomeruli, glomerulosclerosis was observed in 1 glomerulus, while the other glomeruli showed glomerular mesangial cell and stroma mild hyperplasia. The capillary loops were open, with a stiff appearance. The basement membrane was thickened, with a small amount of spike-like structures visible. Subepithelial deposition of immune complexes was observed. No cellulose like necrosis, platinum ear-like structure or crescent formation was observed. The following changes were observed: granular and vacuolar degeneration of renal tubular epithelial cells, occasional protein tubular type, focal renal tubular lumen dilation accompanied by segmental epithelial cell detachment, brush border detachment, renal tubular atrophy, renal interstitial edema, small focal lymphoid and monocyte infiltration. There was no obvious lesion on the small artery wall.

**Figure 2:**
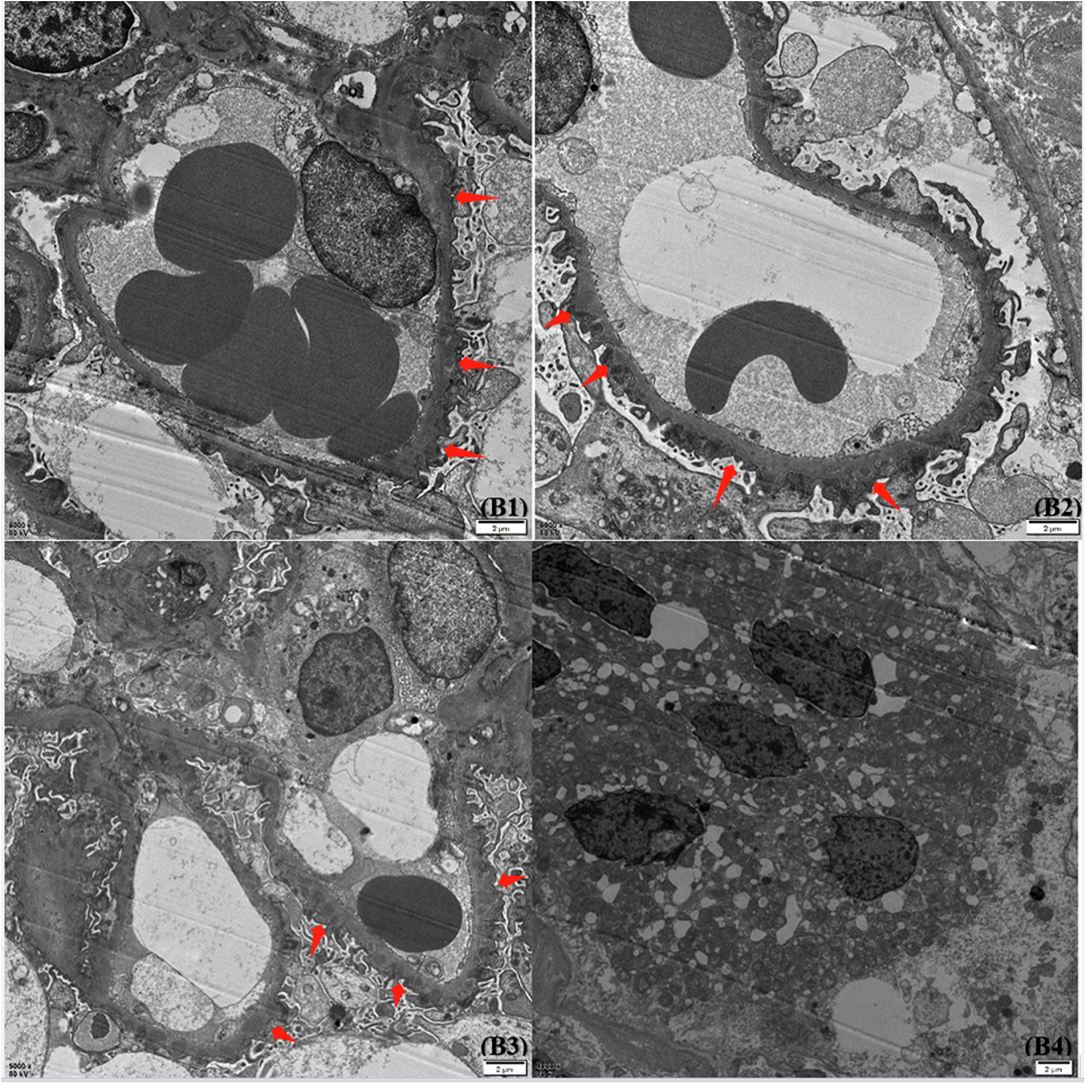
Glomerular findings by electron microscope. Under an electron microscope, the basement membrane showed mild irregular thickening, with a thickness of about 300–700 nm. The foot processes were diffusely fused, and a large amount of electronic dense material was deposited in the subepithelial and basement membranes. The epithelial cells of the renal tubules were vacuolated and degenerated, and there were no special changes in the renal interstitium.

**Figure 3:**
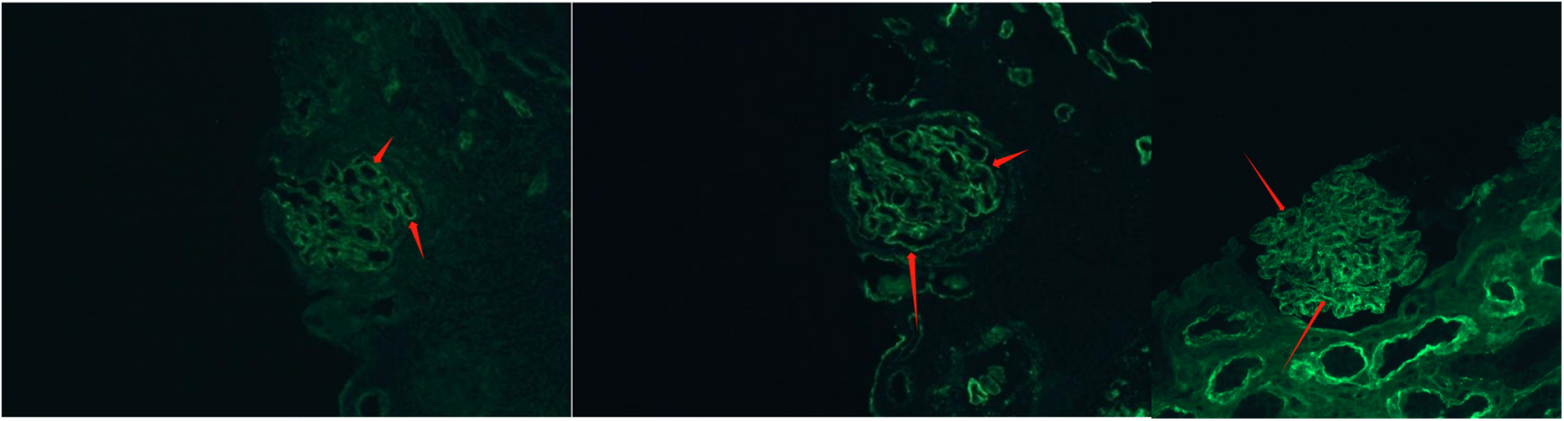
Glomerular findings by immunofluorescence staining. Epidemic immunofluorescence staining showed IgG deposits (++) in fine granular form along the capillary loop.

A 4-year- and 8-month-old boy was brought to our hospital with fatigue, polyuria, and excessive drinking for one month. There was no recent history of fever, hair loss, facial erythema, oral ulcers, vomiting or diarrhea. There was no consanguineal marriage history, no family history of kidney disease and other genetic diseases. He was born spontaneously at 39 weeks, weighed 2.8 kg at birth, was 50 cm long, and had no history of asphyxia (Apgar score unknown). The family thought that he was shorter than other children of the same age and gender, but he was not lagging behind in motor, language and intellectual development. At the age of 1, he was 72 cm tall (−2SD∼−1SD) and weighed 8.5 kg (−2SD∼−1SD); he was 103 cm tall and weighed 12 kg at the time of consultation, and his physical examination height and weight were less than 2 standard deviations of the standard value, his intellectual development was normal, and he could talk and express needs normally. Blood pressure was normal, and there was no edema throughout the body except a ∼3 cm × 3 cm area of cafe au lait spoton the skin of the left lower limb. No other abnormalities were found on physical examinations. Urinary examination showed alkaline urine (urine pH >5.5), glycosuria and highly positive proteinuria, but no hematuria was detected by high-power microscopy. Blood gas analysis and biochemical tests indicated metabolic acidosis and electrolyte disorders, mainly manifested as hyponatremia, hypokalemia, hypocalcemia, hypophosphatemia, and hyperchloremia. The complication of Fanconi syndrome was considered. Further, we carried out humoral immunity test which revealed a decrease in complement C3 (0.38 g/L; the levels of C3 with the normal range was 0.70–2.06 g/L), and the complement C4 was normal (the normal range of C4 level was 0.11–0.61 g/L). Autoantibody ANA was positive, with the highest titer >1:3,200. Thyroid function was normal. Recent laboratory results were shown in Table 1. Urinary ultrasound indicated enhanced renal parenchymal echoes with unclear corticomedullary boundary, hydronephrosis in both kidneys, and no renal calcification or stones. The bone age was relatively below the standard, equivalent to 3.6-year-old or 10–25 percentiles. The contrast-enhanced voiding urosonography (VUS), CT urography (CTU), axial enhanced MRI of the sella turcica, conventional MRI of the lower limbs, and electromyography examination revealed no abnormalities. The whole-exome sequencing of the child did not found any suspected pathogenic gene mutations. After excluding diseases such as tuberculosis, hepatitis B, diabetes, genetic diseases and tumors, the patients were treated with sufficient oral methylprednisolone, captopril, potassium citrate, sodium citrate, potassium dihydrogen phosphate, and sodium dihydrogen phosphate for 28 days, but there was no improvement in proteinuria. Then renal biopsy was performed, and hematoxylin-eosin and other specific staining revealed 1/4 glomerular glomerulosclerosis. The remaining glomeruli showed mesangial cell and stroma mild hyperplasia, with thickening of the basement membrane and a small amount of spike like structures. Subepithelial deposition of immune complexes was observed. The staining also revealed granular and vacuolar degeneration of renal tubular epithelial cells, occasional protein tubular type, focal renal tubular lumen dilation accompanied by segmental epithelial cell detachment, brush border detachment, a few renal tubular atrophy, renal interstitial edema, small focal lymphoid and monocyte infiltration. There was no obvious lesion on the small artery wall. Immunofluorescence showed that IgG was deposited (++) in fine particles along the capillary loop. The electromicroscopic examination of ultrastructures showed mild irregular thickening of the basement membrane with a thickness of about 300–700 nm, diffused fusion of the foot processes, deposition of a large amount of electronic dense materials in the subepithelial and basement membranes, vacuolization and degeneration of the epithelial cells of the renal tubules, and no special changes in the renal interstitium. The pathological diagnosis was stage II membranous nephropathy with acute tubulointerstitial lesions. Paraffin section fluorescence staining showed IgG1 subtype+, IgG4++, while negative in IgG2, IgG3, PLA2R, and THSD7A staining, consistent with stage II membranous nephropathy (Figures 1–3). Clinical diagnosis of lupus nephritis (nephrotic syndrome type, V-type) was made, with a SLE disease activity index (SLEDAI) score of 14. After receiving a sufficient oral dose of methylprednisolone (12 mg, bid) for 60 days with the subsequent dosage tapered, and sequential anti-inflammatory treatment with prednisone acetate (10 mg, qd), immune suppression with mycophenolate mofetil (0.166 g, bid), 5 times of plasma exchange, captopril, and dipyridamole, the patient was discharged. A follow-up after taking the above oral drugs for 11 months showed that the symptoms of fatigue, excessive drinking, and polyuria improved, the complement C3 returned to normal levels, but there were still persistent proteinuria (2 + to 3+) and glycosuria (2 + to 4+); no hematuria was detected under high-power microscopy; SLEDAI score was 12; autoantibody ANA was positive with a titer of 1:320; and eGFR was calculated to be >30 ml/min/1.73 m^2^ (The eGFR at initial presentation was 53.1 ml/min/1.73 m^2^). Then the treatment with belimumab monoclonal antibody was added. After 9 times of regular use of belimumab, the clinical symptoms of the patient achieved complete remission; blood electrolytes, complement C3 and C4 returned to normal levels; and ANA titer was maintained at a relatively low level (1:320); urinary protein fluctuated between+to ∼+; urinary glucose fluctuated between+to 3+, the UTP was 0.785 g/24 h (24 h urine output of 1,700 ml), and eGFR was calculated to be 32.8 ml/min/1.73 m2, recent laboratory results were shown in Table 1. The dose of prednisone was reduced to 7.5 mg, qd. The follow-up examination showed a body-weight increase to the 50th percentile of the same age and gender, a height of the 3th percentile, and a SLEDAI score decrease to 4.

**Table 1:** Laboratory results.

	2021-04-24 (At presentation)	2022-04-28 (Before belimumab)	2023-5-23 (After treatment)
Blood pH	7.21	7.40	7.33
Blood PCO2 (mmHg)	17.00	33.75	47.25
Serum sodium (mmol/L)	131.00	137.00	141.7
Serum potassium (mmol/L)	2.60	3.70	3.83
Serum calcium (mmol/L)	1.69	2.35	2.41
Serum chlorine (mmol/L)	99.00	108.00	113.20
Serum phosphate (mmol/L)	0.67	1.30	1.35
Serum creatinine (umol/L)	73.00	74.00	145.00
Serum albumin (g/L)	31.50	45.50	45.60
Urine routine	Sugar (+++), protein (++), pH 7.5, specific gravity 1.004	Sugar (+++), protein (++), pH 7.0, specific gravity 1.018, renal intratubular casts (+)	Sugar (+), protein (+-), pH 7.5, specific gravity 1.017
UTP (g/24 h)	1.499 (24 h urine output of 2,200 ml)	0.333 (24 h urine output of 600 ml)	0.785 (24 h urine output of 1,700 ml)
C3 (g/L)	0.38	0.80	0.80
C4 (g/L)	0.20	0.18	0.26
ANA	>1:3,200	1:320	1:320
ENAs	Negative	Negative	Negative

## Discussion

SLE is a relatively rare systemic autoimmune disease. If not treated in a timely manner, irreversible damage to various organs can occur, ultimately leading to death (10). Childhood SLE refers to SLE that starts before the age of 18 years, accounting for 1/5 of all SLE cases (10). The onset age of cSLE in Asia is 8.6–13.5 years old, with only 5% of cases occurring before the age of 5 years (11). Compared with adult SLE, cSLE may have a higher correlation with genetic susceptibility, and cSLE has a more acute onset and more severe symptoms, with a higher probability of developing proteinuria, facial erythema, anti-dsDNA antibodies, hemolytic anemia, arthritis, etc. (12), Which further increasing the disability and mortality rate of the disease, lupus nephritis is one of the main causes of death in cSLE patients, 50%–70% of cSLE children can develop lupus nephritis (13–15), ranging from asymptomatic hematuria and/or proteinuria to rapidly progressive nephritis with renal function impairment, nephrotic syndrome, and end-stage kidney disease.

The clinical manifestations and initial symptoms of cSLE vary. About 60%–85% of children with SLE have atypical onset symptoms (3, 4). The onset age of the present case was young, and the symptoms were extremely atypical. The onset was characterized by fatigue, polydipsia, and polyuria. Combined with persistent metabolic acidosis and urinary pH >5.5, the patient was initially diagnosed with type I renal tubular acidosis (distal renal tubular acidosis, dRTA). Further complication was Fanconi syndrome based on the patient's normal blood glucose levels, sustained glycosuria, developmental delay, polyuria, thirst, hypophosphatemia, hypokalemia, metabolic acidosis, etc. Finally, the diagnosis of cSLE was made through renal biopsy. To our knowledge, there has been no report of cSLE combined with Fanconi syndrome. The case we reported was cSLE combined with type I renal tubular acidosis and Fanconi syndrome, which has not been previously reported.

SLE related kidney injuries may involve the glomerulus, tubulointerstitium, and vascular endothelium, but the involvement of the latter two have been rarely reported (16). Nowadays, the concept of SLE-associated tubulointerstitial nephritis (SLE TIN) is increasingly being proposed (17). Some scholars have pointed out that tubulointerstitial injury may be related to glomerular disease. At the same time, in the context of severe or active glomerular disease, interstitial changes in the tubules may not be obvious or even absent, or isolated tubular injury may occur in the absence of glomerular derived proteinuria (5, 18). The inconsistent occurrences suggest that there may be independent pathogenesis between tubular injury and glomerular disease (19). Tubulointerstitial injury is of great clinical significance, because it is not only a precursor to the occurrence of lupus, but also an independent predictive indicator of disease prognosis (20–22). SLE with renal tubular acidosis (RTA) has been widely reported. RTA is a kind of disease classified by clinical symptoms and biochemical features, which are characterized by the impairment of bicarbonate reabsorption or hydrogen secretion in different parts of renal tubules, leading to renal acidification dysfunction (23). The pathogenesis of SLE-related RTA is still unclear, which may be related to the deficiency of H^+^ -ATPase in intercalated cells on collecting duct caused by autoantibodies (18). The increase in membrane permeability of renal tubular lumen cells can also lead to the loss of a large amount of potassium ions along the concentration gradient, thereby inhibiting H^+^ -ATPase activity and leading to the disease development. Previous studies found that the presence of autoantibodies targeting carbonic anhydrase-II in patients with tubulointerstitial nephritis was associated with type-1 and type-3 RTA (15). Li et al. reported that 6 adult SLE patients were diagnosed with RTA in the later stage of SLE, with an average interval of 3 years between the two diagnoses; among the patients, 5 (83.3%) had proteinuria, and 1 (16.7%) had no significant proteinuria (14). Bagga and Nandi et al. reported that two children with RTA were initially diagnosed, but after targeted therapy, their conditions did not improve; the cause was repeatedly searched, and ultimately the patients were diagnosed with cSLE through renal tissue biopsy and strong serological evidence (24, 25). RTA may be the first symptom of SLE or may appear after diagnosis of SLE. RTA may be present from 1 month to 8 years after the onset of SLE, and fatigue and polyuria are common complaints in these children (14, 25). Renal biopsy of cSLE with RTA often show glomerular tissue hyperplasia, sclerosis, immune complex deposition, renal tubular atrophy, interstitial inflammation and fibrosis formation, consistent with our report. Among the abnormalities, tubulointerstitial lesions are considered as a strong predictor of renal function impairment and unfavourable long-term renal outcomes in LN (17, 21, 22, 26–29).

We searched the literature and found 7 case reports of cSLE combined with renal tubular acidosis, including 6 cases of cSLE combined with type 1 (distal type) renal tubular acidosis (24, 25, 30–33), and 1 case with unknown type of renal tubular acidosis (34). The basic clinical characteristics are shown in Table 2. These reports showed that SLE was often associated with type 1 renal tubular acidosis, with type 4 being rare, and type 2 (proximal type) even more rare (14, 17). In addition, previously reported cases of SLE combined with RTA were more common in other overlap syndromes, where these patients also had other immune diseases, such as autoimmune thyroiditis and Sjogren's syndrome (18, 35).

**Table 2:** Baseline characteristics of diagnosed of cSLE and dRTA.

First author	Year	Country	Sex	Age at onset	Clinical symptoms	Diagnosis	Autoantibodies	Glomerulo-pathy	Types of renal tubular acidosis	Gene	Follow-up
Agrwal (30)	2019	India	F	14-years	Poor growth, polyuria, polydipsia and bony deformities of lower limbs	dRTA, SLE	ANA positive	None	dRTA	NC	Steroid was tapered to a low dose at the end of 12 weeks. She did not develop any proteinuria or hematuria till now.
Bagga (24)	1993	India	F	7-years	Episodic muscular weakness with inability to stand and walk	SLE with diffuse proliferative and sclerosing glomerulonephritis	ANA positive	Diffuse proliferative glomerulonephritis	dRTA	NC	Increased glomerulosclerosis after 1 year
Barathidasa (31)	2020	India	F	8-years	Pyrexia of unknown origin in association with pallor and arthralgia	SLE	ANA positive and anti-dsDNA positive	IV nephritis	dRTA	NG	Lupus nephritis was in remission
Fortenberry (32)	1991	USA	F	14-years	Nausea, vomiting, anorexia, malaise, and weight loss	SLE	ANA positive	Moderate focal tubulointerstitial inflammation	dRTA	NC	Free of active renal disease for the past 18 months
Hataya (33)	1999	Japan	F	12-years	Fever and diarrhea	SLE	ANA positive and antinative DNA positive	Focal mesangial cell proliferation	dRTA	NC	Unclear
Nandi (25)	2016	India	F	9-years	Not gaining adequate height and weigh, anorexia, weakness	SLE	ANA positive and anti-dsDNA positive	Unclear	dRTA	NC	Improved clinically, bio-chemically, and serologically
Park (34)	2014	Korea	F	12-years	Dizziness	SLE combined with hemolytic anemia and renal tubular acidosis	Unclear	Unclear	Unclear	NC	Anemia and renal tubular acidosis were fully recovered after 15 months

ANA, anti-nuclear antibody; dRTA, distal renal tubular acidosis; F, female; NC, unclear; NG, negative; SLE, systemic lupus erythematosus; Y, year.

Fanconi syndrome (FS), also known as Fanconi-Debré-de Toni syndrome or osteomalacia-renal glycosuria-aminoaciduria-hyperphosphateuria syndrome. FS can be congenital or acquired, transient or persistent, and can be accompanied by end-stage renal disease or a normal glomerular filtration rate (36–38). Previously, it was believed that the disease was hereditary, but in recent years, multiple clinical studies have suggested that its etiology is mostly acquired (39). Due to atypical symptoms and diverse causes, it is highly susceptible to misdiagnosis. Secondary FS is often secondary to immune diseases such as Sjogren's syndrome, multiple myeloma, anaphylactoid purpura, and can also be secondary to metabolic diseases such as galactose, hepatolenticular degeneration, or exposure to drugs and toxins that induce mitochondrial damage in kidneys (40, 41). cSLE combined with renal tubular acidosis often involves the distal renal tubules, yet the occurrence of Fanconi syndrome would indicate proximal renal tubular damage. This type of complication has not been previous reported. In our case, the renal pathology revealed stage II membranous nephropathy with acute tubulointerstitial injury. The pathogenic immune mechanism of SLE remains unclear, and the mechanism of proximal or distal tubular epithelial cell injury caused by lupus nephritis remains unclear. Some studies have shown that over-absorption of IgG anti-dsDNA antibodies and albumin by proximal tubular epithelial cells results in ROS-mediated tubular injury and interstitial inflammation (42). Fanconi syndrome may be caused by extensive proximal convoluted tubule injury caused by the disease. In our case, tubular injury was found in the kidney biopsy and renal intratubular casts were found in the urine routine, indicating this possibility. In addition, we suspected that most children with lupus nephritis do not have Fanconi syndrome, which may be related to the strong compensatory capacity of the glomeruli in children and the lack of serious renal interstitial damage. The severity of proximal renal tubule injury may be the key condition for the occurrence of typical Fanconi syndrome, but the specific mechanism needs further study.

The case reported here was prone to misdiagnosis in clinical practice, since the age of the child was not within the typical age range for childhood SLE, and the clinical symptoms were extremely atypical. If the renal biopsy findings were unavailable, the clinical manifestations would not meet the diagnostic criteria for cSLE, and the symptoms of the child such as fatigue significantly improved after the treatment for renal tubular acidosis, making it easy to misdiagnose. This patient had refractory hyperchloremic metabolic acidosis, hypokalemia, alkaline urine (urine pH >5.5), and persistent proteinuria, as well as manifestations of renal glycosuria, proteinuria, refractory electrolyte disorders, growth and development retardation combined with Fanconi syndrome. Before treatment, we conducted detailed medical history inquiry, physical examination and examination of the child. There were no patients with long-term chronic kidney disease in his family, and the simple examination of urine and kidney function of the parents of the child showed no abnormalities. In addition, the whole exon sequencing test showed no suspected pathogenic gene variation, and the regular child health examination in the local hospital before the child was 3 years old showed no obvious abnormalities, so the possibility of FS caused by congenital or genetic factors were not supported. Acquired FS usually occurs secondary to diseases or exposure to certain toxins or drugs, but a detailed history did not reveal the consumption of any potentially nephrotoxic medications or exposure to radiation or environmental toxin. Furthermore, the patient was not complicated with Henoch–Schonlein purpura, liver disease, diabetes, thyroid disease, chronic infection, tumor, or metabolic disease, and there was no strong evidence to support obstructive nephropathy, so it was highly suspected that cSLE lesions involved renal tubules combined with acquired FS.

However, it was questionable why the child has significant growth delay and a decrease in eGFR at the time of presentation. On the one hand, we believed that the symptoms of SLE in children were too insidious, resulting in family members not finding abnormalities in time, and the child was too young to accurately explain his physical discomfort. On the other hand, kidney damage caused by the disease may be compensated by a subset of normal nephrons, so symptoms were masked. Based on the above two aspects, it is speculated that the child may have a longer course of disease than the chief complaint, so there has been significant growth retardation and decline in kidney function at the time of presentation. The above conclusions further indicate that atypical cSLE is difficult to detect due to insidious symptoms, and delayed medical treatment may cause irreversible damage to kidney function, so doctors need to be more sensitive to find the problem and diagnose it in time.

LN is by default severe disease, that can lead to gradual nephron loss and chronic kidney disease (CKD) in addition to death. belimumab is currently the only biological agent approved for the treatment of cSLE, achieving therapeutic effect primarily by binding to B-lymphocyte activating factors (43). In a post-hoc analysis of the BLISS-LN, belimumab was found to reduce the risk for flares by 55% compared with standard-of-care (SoC, low-dose CYC62 or mycophenolate in combination with GC) alone, and preserve glomerular filtration rate (GFR) better than SoC (44). Belimumab may be considered as an add-on therapy in patients with refractory or relapsed SLE to reduce disease activity, disease recurrence rate, and hormonal dose. But the current recommendations do not require prior failure to one or more conventional drugs before initiating a biological agent. It is worth noticing that after nearly a year of combined treatment with immunosuppressive agents and corticosteroids, the patient's high levels of proteinuria and glycosuria were not corrected satisfactorily. Considering that the pathological type of the patient's lupus nephritis was nephrotic syndrome type (V-type) with renal tubulointerstitial injury, a case of refractory lupus nephritis. After fully communicating with the child's family and obtaining their consent, we added the biological agent belimumab into the therapy. The patient's clinical symptoms, such as fatigue, were completely relieved after 9 times of regular use of belimumab. In addition, the patient's blood electrolytes, complement C3 and C4 returned to normal levels; ANA titer decreased to 1:320; urinary protein fluctuated between urinary glucose fluctuated between + to ∼+; urinary glucose fluctuated between + to 3 + . The dose of prednisone was smoothly tapered to 7.5 mg, qd. The patient's body-weight increased to the 50th percentile of the same age and gender; his height was in the 3th percentile; and SLEDAI score decreased to 4 on the most recent follow-up. Currently, his 24-hour urinary protein is close to a normal level. These results suggest that belimumab is effective in the treatment of the patient's disease.

In conclusion, the clinical manifestations of SLE in children can be very insidious, and tubular acidosis may be the first and prominent manifestation of childhood lupus nephritis. So far, there has been no report of secondary Fanconi syndrome in children with lupus nephritis. Therefore, for children with symptoms such as unexplained renal tubular acidosis, hypokalemia, proteinuria, and renal glycosuria, it is necessary to be alert to atypical cSLE in addition to congenital and genetic metabolic diseases. It is recommended to carry out long-time follow-ups, screen for autoantibodies and perform other immune tests in a timely manner. When a clinical diagnosis is difficult, a clear diagnosis can be made through renal puncture biopsy, so that the patient can be diagnosed and treated early with an improved prognosis.

## Data Availability

The raw data supporting the conclusions of this article will be made available by the authors, without undue reservation.
